# Casein Kinase II Inhibitor Enhances Production of Infectious Genotype 1a Hepatitis C Virus (H77S)

**DOI:** 10.1371/journal.pone.0113938

**Published:** 2014-12-02

**Authors:** Seungtaek Kim, Bora Jin, Sung Hoon Choi, Kwang-Hyub Han, Sang Hoon Ahn

**Affiliations:** 1 Severance Biomedical Science Institute, Yonsei University College of Medicine, Seoul, Korea; 2 Institute of Gastroenterology, Department of Internal Medicine, Yonsei University College of Medicine, Seoul, Korea; 3 Brain Korea 21 Plus Project for Medical Science, Yonsei University College of Medicine, Seoul, Korea; Rutgers, The State University of New Jersey, United States of America

## Abstract

Genotype 2a JFH1 virus has substantially contributed to the progress of HCV biology by allowing entire viral life cycle of HCV in cell culture. Using this genotype 2a virus, casein kinase II (CKII) was previously identified as a crucial host factor in virus assembly by phosphorylating NS5A. Since most of the prior studies employed genotype 2a JFH1 or JFH1-based intragenotypic chimera, we used genotype 1a H77S to study virus assembly. CKII inhibition by chemical inhibitors enhanced H77S virus production in contrast to that of JFH1 virus, but genetic inhibition of CKII by siRNA did not change H77S virus titer significantly. The different outcomes from these two approaches of CKII inhibition suggested that nonspecific target kinase of CKII inhibitors plays a role in increasing H77S virus production and both viral and host factors were investigated in this study. Our results emphasize substantial differences among the HCV genotypes that should be considered in both basic research and clinical practices.

## Introduction

Hepatitis C virus (HCV) is a causative pathogen of chronic hepatitis C, cirrhosis, and hepatocellular carcinoma and approximately 170 million people are infected worldwide with this virus (for a review, see [1]). Although there has been a substantial progress in the development of interferon-free, all-oral antiviral regimens, still many people are suffering from these deadly viral diseases. Specifically, infection with genotype 1a HCV, previous null response to pegylated interferon-α/ribavirin therapy, and cirrhosis are difficult cases to cure [2].

HCV belongs to the *Hepacivirus* genus within the *Flavivirida*e family and has a positive-sense, single-strand RNA (9.6 kb) as its genome. A single polyprotein translated from this viral RNA is processed co- and post-translationally by host and viral proteases to generate 10 viral proteins. Core, E1, and E2 proteins located at the N-terminus of the polyprotein are structural proteins and components of virus particles. The other proteins (p7, NS2, NS3, NS4A, NS4B, NS5A, and NS5B) located at the C-terminus of the polyprotein are nonstructural proteins and participate in diverse steps of viral life cycle including genome replication, particle assembly, etc. Of these, the proteins from NS3 to NS5B are sufficient for viral RNA replication as members of replication complex [3] and in this complex, NS5B functions as RNA-dependent RNA polymerase (RdRp). Since JFH1 and H77S were discovered as cell culture infectious HCV clones [4], [5], studying all steps of HCV viral life cycle has become possible and novel functions of nonstructural proteins in HCV life cycle other than viral RNA replication have been intensively studied (for a recent review, see [6]).

Post-translational modification such as phosphorylation plays a crucial role in many steps of viral life cycle including HCV. Specifically, phosphorylation of NS5A has been considered as a molecular switch determining the role of NS5A between viral RNA replication and particle assembly [7], [8], and the status of phosphorylation is displayed as differentially phosphorylated NS5A species (56kDa basal phosphorylation and 58kDa hyper-phosphorylation). Recently, some specific serine and threonine residues of NS5A were identified as phosphorylated amino acids by mass spectrometry [9], [10]. Also, Tellinghuisen et al. [11] uncovered a novel role of casein kinase II (CKII) in HCV infectious particle assembly, which phosphorylates a single serine residue located at the C-terminus of NS5A domain III although direct biochemical evidence of such phosphorylation has not been provided yet. In their study, treatment of HCV RNA-transfected cells with 2-dimethylamino-4,5,6,7-tetrabromo-1*H*-benzimidazole (DMAT), a CKII inhibitor, reduced virus production without affecting viral RNA replication and the similar result was reproduced with knockdown of CKII by siRNA [11]. Thus, CKII inhibitor could be considered as another host-targeting antiviral therapeutic option, specifically inhibiting infectious particle assembly of HCV. In fact, CX-4945, a selective CKII inhibitor, has entered human clinical trials although it was for its anti-tumor activity not for antiviral activity [12].

There are 7 major genotypes of HCV [13] and the pairwise differences of nucleotide sequences between the genotypes are on the order of 31 to 33% due to the error-prone NS5B RNA-dependent RNA polymerase. Differences of sequences among the genotypes are also reflected in the response to interferon-α-based antiviral treatment. For example, the treatment with pegylated interferon-α and ribavirin achieved 76–82% of sustained virologic response (SVR) in genotype 2 and 3 patients while it achieved only 42–46% of SVR in genotype 1 patients [14], [15]. Even with several direct-acting antivirals (DAAs), the treatment response is dependent on the genotypes of HCV [2], thus the identification of genotype is still very important in selecting treatment options and predicting treatment outcomes of HCV patients.

In this study, we tested whether treatment of CKII inhibitor could reduce virus production of genotype 1a HCV as efficiently as genotype 2a virus. Although many significant findings were made possible due to the development of genotype 2a JFH1 infectious clone [4], direct application of such findings in clinical trials should await further validation especially in genotype 1a cell culture system considering the aforementioned significant differences among the HCV genotypes.

## Materials and Methods

### Plasmids

Most of the plasmids in this study have already been described [16], [17] except for JFH1/H3 (1a/2a intergenotypic chimera containing NS3 of H77S.3 in JFH1 background), JFH1/H4AB (1a/2a intergenotypic chimera containing NS4AB of H77S.3 in JFH1 background), H77S.3/J5B (1a/2a intergenotypic chimera containing NS5B of JFH1 in H77S.3 background), and JFH1/H5B (1a/2a intergenotypic chimera containing NS5B of H77S.3 in JFH1 background). JFH1/H3 and JFH1/H4AB were constructed by ligating DNA fragments generated from EcoRI/NotI digestion of pJFH1/H3/GLuc2A and pJFH1/H4AB/GLuc2A [18] in the vector plasmid pJFH1. NS5B swap mutants, H77S.3/J5B and JFH1/H5B were constructed by ligating DNA fragments generated from NsiI/XbaI digestion of pH77S.3/J5B/GLuc2A and pHJ3-5/H5B/GLuc2A [18] in the vector plasmids pH77S.3 and pJFH1, respectively. Mutated sequences were verified by restriction analysis and DNA sequencing analysis.

### Cells

Huh7.5 cells [19] were used for all the experiments in this study. The cells were maintained in DMEM high glucose medium containing 10% fetal bovine serum and 1X penicillin/streptomycin at 37°C in a 5% CO_2_ environment.

### RNA transcription and transfection

Plasmid DNAs were linearized by XbaI restriction digestion before in-vitro transcription reaction. RNAs were then synthesized from the linearized DNAs using MEGAscript kit (Ambion). The transcribed RNAs were confirmed by spectrophotometer and electrophoresis. One day before transfection, Huh7.5 cells were seeded in 6-well culture dishes (6×10^5^ cells/well). In-vitro transcribed RNAs were transfected by TransIT-mRNA transfection kit (Mirus Bio), and 6 hours after transfection, the transfected cells were split by a 1∶2 ratio.

### DNA transfection

Plasmid DNAs were transfected by TransIT-2020 reagent (Mirus Bio) as recommended by the manufacturer's instruction.

### siRNA transfection

siRNAs for CKIIα (#1337) and CKIIα′ (#183) were purchased from Ambion. These siRNAs were transfected by TransIT-TKO transfection reagent (Mirus Bio) as recommended by the manufacturer's instruction. The final concentration of siRNA was 50nM. For transfection of both HCV RNA and siRNA in the same cells, HCV RNA was transfected first, and 6 hours later, the cells were washed by PBS and transfected with siRNA.

### Virus titration

Huh7.5 cells were seeded a day before infection in 48-well culture dishes (1×10^5^ cells/well). Culture supernatant collected from the RNA-transfected cells at day 3 after transfection was added to the naïve cells for infection. Three days after infection, the cells were fixed and HCV core protein was labeled as described [20]. The number of infected foci was counted manually under the fluorescence microscope.

### GLuc reporter assay

Culture supernatant from the *Gaussia* lucifease sequence-containing RNA-transfected cells was collected daily to measure secreted GLuc activity using BioLux *Gaussia* Luciferase Assay Kit (New England BioLabs) as was described [21].

### CKII inhibitor treatment

Six hours after HCV RNA transfection, the transfected cells were split by a 1∶2 ratio, and refed with fresh medium containing 2-dimethylamino-4,5,6,7-tetrabromo-1*H*-benzimidazole (DMAT) or (*E*)-3-(2,3,4,5-tetrabromophenyl)acrylic acid (TBCA), specific CKII inhibitors (Calbiochem). The cells were incubated for 48 hours prior to being refed with fresh medium (without inhibitors). Culture supernatant fluids were collected one day later and used for the titration of infectious viruses. Cytotoxic effects of DMAT were assessed using the WST-1 Cellular Proliferation Assay (Roche Applied Sciences) as recommended by the manufacturer.

### Immunoblot

A standard immunoblot procedure was employed [22]. Protein samples transferred to PVDF membranes were probed with the following primary antibodies: anti-core (1∶2,000, Affinity BioReagents, MA1-080), 9E10 (kindly provided by Dr. Charles Rice and Dr. Tim Tellinghuisen), anti-NS3 (1∶1,000, Virogen, 217-A), anti-CKIIα (1∶2,500, Bethyl Laboratories, A300-197A), anti-CKIIα′ (1∶2,500, Bethyl Laboratories, A300-199A), and anti-GAPDH (1∶500,000, Ambion, AM4300) antibodies. Proteins were visualized with IRDye 800CW Goat anti-Mouse IgG or IRDye 680 Goat anti-Rabbit IgG, and images collected on an Odyssey infrared imaging system (LI-COR Biosciences).

## Results

### DMAT increases virus production of genotype 1a HCV

**Figure 1:**
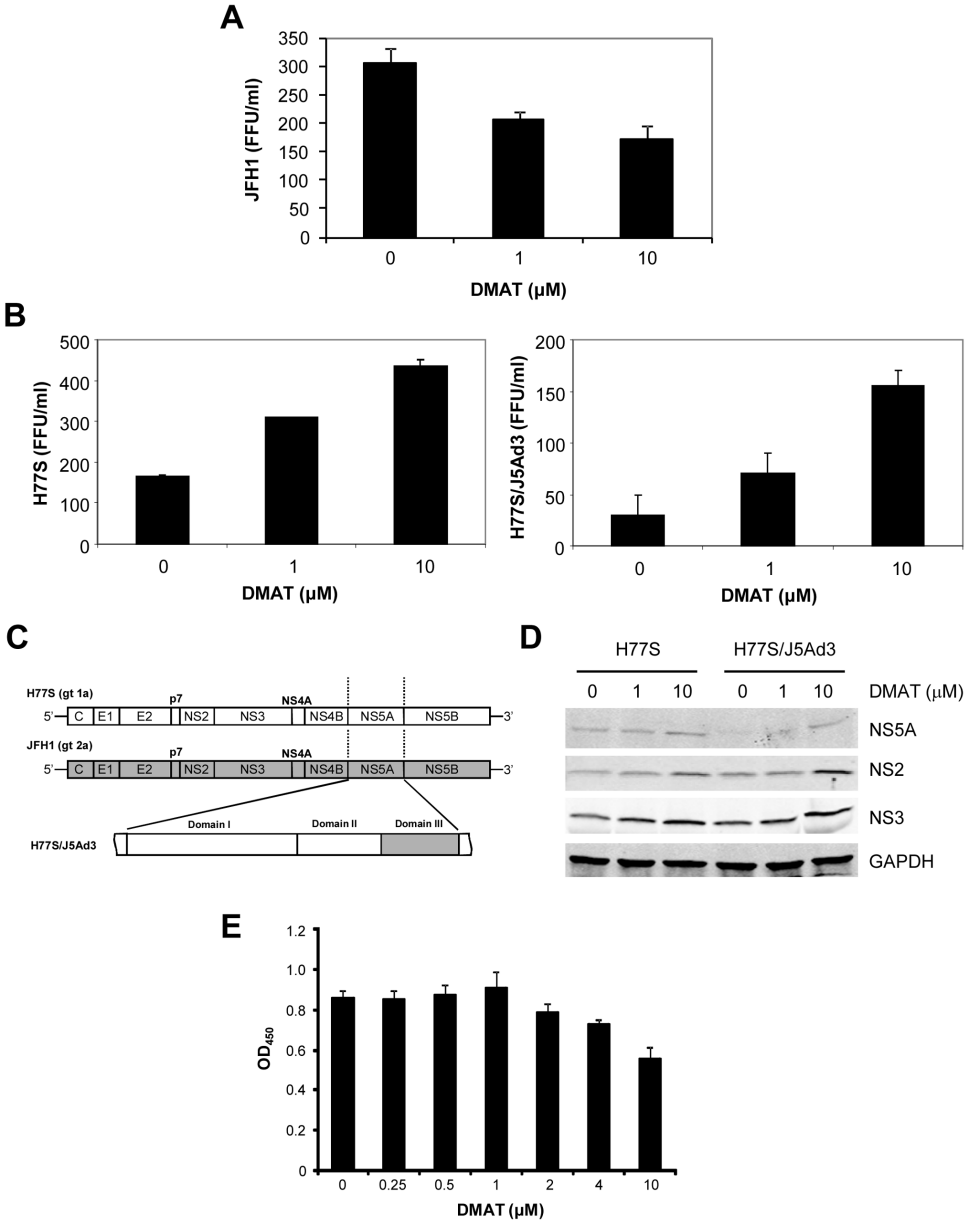
Enhanced H77S virus production by DMAT, a CKII inhibitor. (A) Following transfection of the JFH1 RNA, cells were treated with the indicated concentration of DMAT for 48 hours. The media was then replaced with fresh medium (no drug), followed 24 hours later by harvesting of supernatant fluids for virus titration. Means ± S.E. were calculated from duplicate experiments. (B) The effect of DMAT treatment on the production of H77S and H77S/J5Ad3 infectious particles. (C) Schematic diagram of the virus for this study. (D) Immunoblots for NS5A, NS2, NS3, and GAPDH from the cell lysates prepared 72 hours after transfection. (E) Cytotoxicity was tested by WST-1 assay. Means ± S.E. were calculated from triplicate experiments.

DMAT, a CKII inhibitor, is known to decrease J6/JFH1 virus production in a dose-responsive manner [11]. We confirmed the similar result with JFH1 virus (Fig. 1A) and its NS5A domain III chimera (JFH1/H5Ad3) in our prior study [17]. Since these results were obtained only from genotype 2a viruses, we further tested DMAT in other HCV viruses to see whether this decrease of virus production is common to other genotypes and chimeras. Huh7.5 cells were transfected by HCV RNA and 6 hours after transfection, DMAT was added to the culture medium and maintained for 48 hours. Three days after transfection, cell lysates and culture supernatants were collected for immunoblot and virus titration, respectively. Surprisingly, the same DMAT treatment rather increased virus production of genotype 1a H77S virus in a dose-responsive manner (Fig. 1B) and a similar result was also observed for its NS5A domain III chimera (H77S/J5Ad3) (see Fig. 1C for the structure of the chimera). Identity of NS5A domain III does not appear to be a critical factor for this outcome (compare H77S and H77S/J5Ad3), as this has already been demonstrated when JFH1 and JFH1/H5Ad3 were compared [17]. Rather, sequence(s) outside of NS5A domain III seems to be a main factor affecting this DMAT treatment.

Increase of virus production was accompanied by increase in abundance of viral proteins. Since NS2 and NS5A are the known substrates of CKII phosphorylation [23], [24], we probed these proteins by immunoblot (Fig. 1D). We found that abundance of both NS2 and NS5A increased when the concentration of DMAT increased. For NS2 of genotype 1a virus, it is already known that NS2 protein becomes unstable upon phosphorylation by CKII [23], however NS2 of genotype 2a virus is relatively stable upon DMAT treatment [17]. Thus, increase of NS2 protein abundance was expected by inhibition of CKII in our experiment. The result from NS5A was contrary to our expectation. Our prior study [17] with Ser-to-Ala and Ser-to-Asp substitution mutants of NS5A domain III of H77S.3 virus (i.e., H77S.3/4SA and H77S.3/4SD) suggested that dephosphorylated form of NS5A could be unstable and do not produce infectious viruses as efficiently as phosphorylated form of NS5A. Thus, our expectation was a reduced abundance of NS5A protein by DMAT treatment. However, we observed an increased abundance of NS5A protein. We also probed the lysates with anti-NS3 antibody as a control since this protein would not be affected by CKII inhibition. Interestingly, abundance of NS3 also slightly increased compared to the GAPDH loading control (Fig. 1D).

The effect of DMAT on cytotoxicity and cell proliferation in Huh7.5 cells was also assessed by WST-1 assay (Fig. 1E). Despite some toxicity in higher concentrations of DMAT (>1 µM) in Huh7.5 cells, we did not observe any apparent difference in cell proliferation between H77S and JFH1 RNA-transfected cells in the presence of CKII inhibitor. Importantly, although the toxicity of DMAT slightly increased when the cells were treated with the inhibitor, H77S virus production rather increased, which is opposite to the well-known negative effect of DMAT on genotype 2a JFH1 virus production.

**Figure 2:**
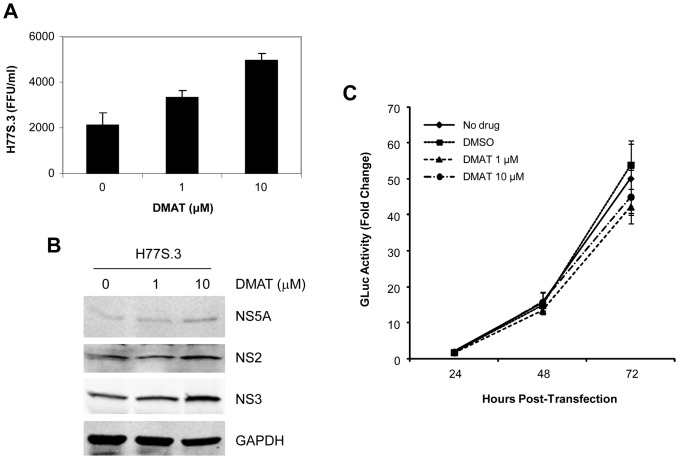
Effect of DMAT on the production of H77S.3 virus. (A) Following transfection of the HCV RNA, cells were treated with the indicated concentration of DMAT for 48 hours. The media was then replaced with fresh medium (no drug), followed 24 hours later by harvesting of supernatant fluids for virus titration. Means ± S.E. were calculated from duplicate experiments. (B) Immunoblots for NS5A, NS2, NS3, and GAPDH from the cell lysates prepared 72 hours after transfection. (C) Effect of different DMAT concentrations on RNA replication measured by GLuc activity secreted from H77S.3 RNA-transfected cells, which contains *Gaussia* luciferase-encoding sequence between p7 and NS2. Means ± S.D. were normalized to the GLuc activity at 8 hours after transfection and calculated from quadruplicate GLuc assays.

Since H77S.3 has a better dynamic range of viral titers, which produces approximately 10-fold more infectious particles compared to H77S [21], we repeated the same experiment with this construct and the result was similar to that of H77S virus both in virus production and protein abundances (Fig. 2AB). We also assessed viral RNA replication by measuring *Gaussia* luciferase activity secreted by H77S.3 RNA-transfected cells, which contains *Gaussia* luciferase sequence between p7 and NS2 (Fig. 2C). Over the 72 hour time-course experiment, no significant difference in GLuc reporter expression was observed among the differentially treated cells. Thus, the enhanced H77S virus production by DMAT treatment appears to be dependent on post-RNA replication step as was the case for J6/JFH1 virus [11] although the effect on virus production was the opposite.

### NS2 and NS5A domain III of genotype 1a HCV

**Figure 3:**
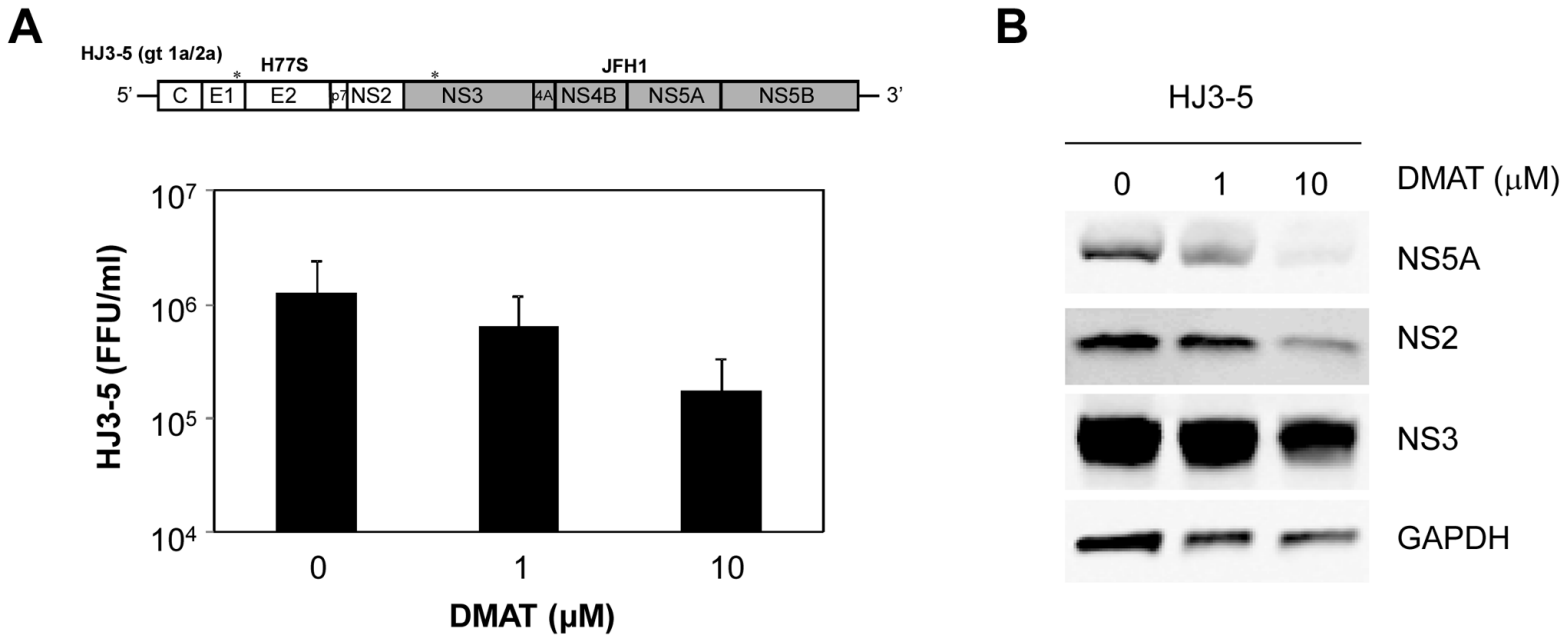
Effect of DMAT on the production of 1a/2a intergenotypic HJ3-5 virus. (A) Following transfection of the HCV RNA, cells were treated with the indicated concentration of DMAT for 48 hours. The media was then replaced with fresh medium (no drug), followed 24 hours later by harvesting of supernatant fluids for virus titration. Means ± S.E. were calculated from duplicate experiments. (B) Immunoblots for NS5A, NS2, NS3, and GAPDH from the cell lysates prepared 72 hours after transfection.

We further tested 1a/2a intergenotypic chimera HJ3-5 [16] virus in the presence of DMAT since this virus contains NS2 from H77S and NS5A from JFH1 virus (Fig. 3A, upper panel). The virus titers decreased when the concentration of DMAT increased (Fig. 3A, lower panel). Immunoblot of the lysates from the transfected cells showed reduced abundance of HCV proteins including NS2, NS5A, and NS3 (Fig. 3B). Overall, the results from HJ3-5 virus were similar to those from JFH1 virus. This outcome is rather surprising because the result is very opposite to that for H77S/J5Ad3 (see Fig. 1B, right panel). Both HJ3-5 and H77S/J5Ad3 contain NS2 from H77S and NS5A domain III from JFH1 (see Fig. 1C and Fig. 3A, upper panel). If there are only two viral factors affected by CKII phosphorylation (i.e., NS2 and NS5A domain III), these two viruses should have the same phenotype upon DMAT treatment, however they did not. Thus, this result suggests that there could be other factor(s) in HCV that is affected by DMAT.

### Effect of DMAT on Ser-to-Ala and Ser-to-Asp substitution mutants of NS5A domain III

**Figure 4:**
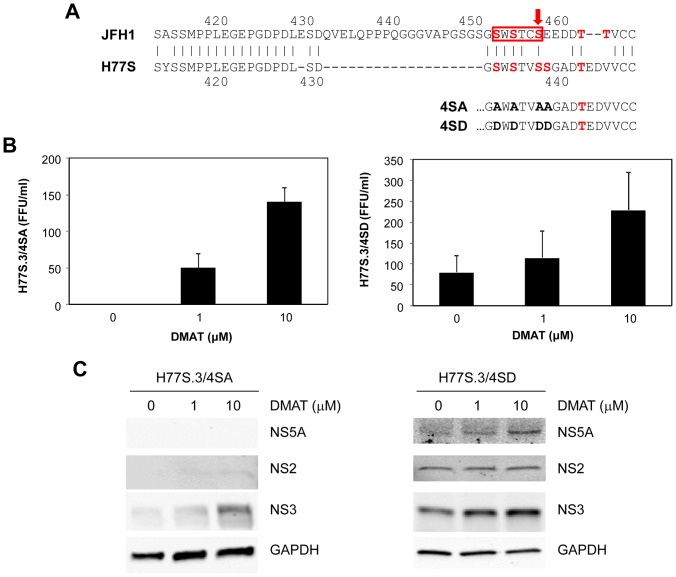
Effect of DMAT on the production of Ser-to-Ala and Ser-to-Asp substitution mutants of NS5A domain III of H77S.3 virus. (A) Possible Ser/Thr phospho-acceptor sites in the C-terminal region of domain III of the NS5A proteins of JFH1 and H77S virus. At the top of the panel, the H77S and JFH1 sequences are aligned: Ser residues found to be important for the NS5A-core interaction and assembly and release of infectious JFH1 virus by Masaki et al. [35] (red box), and Ser-457, identified by Tellinghuisen et al. [11] as a site of CKII phosphorylation (red arrow), are highlighted. Within the related H77S sequence, Ser-438 and Thr-442 are possible sites of CKII phosphorylation predicted by the NetPhos 1.0 server. Below are shown the C-terminal NS5A sequences of 4SA and 4SD substitution mutants. Potential Ser/Thr phospho-acceptor sites are shown in red, while Ala and Asp substitutions in the mutants are shown in bold-face type. (B) Following transfection of the RNA, cells were treated with the indicated concentration of DMAT for 48 hours. The media was then replaced with fresh medium (no drug), followed 24 hours later by harvesting of supernatant fluids for virus titration. Means ± S.E. were calculated from duplicate experiments. (C) Immunoblots for NS5A, NS2, NS3, and GAPDH from the cell lysates prepared 72 hours after transfection.

We also tested our Ser-to-Ala (H77S.3/4SA) and Ser-to-Asp (H77S.3/4SD) substitution mutants of NS5A domain III (Fig. 4A) in the presence of DMAT since these mutated sequences would not be sensitive to the compound. In our prior investigation, we found that 4SA mutant does not produce infectious particles despite comparable RNA replication [17]. However, 4SD mutant partially restored production of infectious viruses. Surprisingly, H77S.3/4SA mutant restored production of infectious particles when the concentration of DMAT increased (Fig.4B, left panel) and it was accompanied by increase in the abundance of NS2 and NS3 proteins. However, NS5A protein could not be still detected by immunoblot (Fig. 4C, left panel). H77S.3/4SD mutant also produced more infectious particles when the concentration of DMAT increased (Fig. 4B, right panel) and the abundance of NS2, NS5A, and NS3 proteins increased concomitantly (Fig. 4C, right panel). The results from these two mutant HCV RNAs suggest that increased virus production of genotype 1a HCV by DMAT is mediated by sequence(s) outside of NS5A domain III sequence. Since the same NS2 of H77S.3 was used for both H77S.3/4SA and H77S.3/4SD viruses, again there seems to be other target(s) of DMAT in HCV in addition to NS2 and NS5A domain III.

The effect of DMAT on the abundance of NS3 of H77S.3/4SA mutant was specifically surprising (Fig. 4C, left panel) since such a substantial increase has never been found in any other mutant constructs. We tested ectopic expression of NS3 in the presence of DMAT by transfecting NS3 and NS3/4A expression plasmids. However, the abundance of NS3 protein decreased when the concentration of DMAT increased (data not shown), thus excluding any stabilizing effect of NS3 protein in the presence of DMAT.

### Knockdown of CKII slightly reduces genotype 1a HCV production

**Figure 5:**
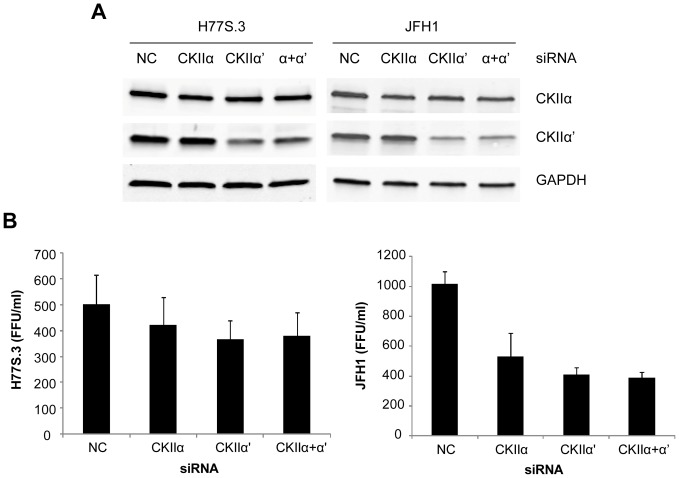
Knockdown of CKII by siRNA trasnfection and its effect on the virus production. Six hours after HCV RNA transfection, cells were transfected with siRNAs for CKII. Three days after transfection, cell lysates and culture supernatants were obtained for immunoblot (A) and virus titration (B), respectively. Means ± S.E. were calculated from triplicate experiments.

All of our experimental data regarding CKII inhibition was so far dependent on chemical inhibitor, DMAT. Since there are numerous examples of nonspecific target effects of chemical inhibitors [25], [26], we tried a genetic inhibition of CKII by siRNA knockdown. Following the method of Tellinghuisen et al. [11], we silenced two catalytic subunits of CKII (α and α′) both individually and simultaneously by siRNAs. In order to test this silencing effect on HCV production, Huh7.5 cells were transfected first with HCV RNA and 6 hours later, the same cells were transfected by siRNA for CKII α and α′. Three days after transfection of both HCV RNA and siRNA, cell lysates and culture supernatants were collected for immunoblot and virus titration, respectively. Fig. 5A shows immunoblot results for CKIIα and CKIIα′. Knockdown of CKIIα′ was better accomplished than that of CKIIα although CKIIα was also reduced approximately by 20∼30% (Odyssey quantitation, data not shown). Accordingly, the effect on virus titration was more substantial with CKIIα′ knockdown in both H77S.3 and JFH1 viruses (Fig. 5B). Interestingly, knockdown of CKII reduced H77S.3 virus production although its negative impact was much smaller than that for JFH1 virus. This result is very opposite to our previous data obtained with DMAT chemical inhibitor (see Fig. 2A). However, the titer of JFH1 virus decreased whether the cells are either treated with DMAT or transfected by siRNA for CKII. The discrepancy between genetic inhibition and chemical inhibition data with H77S.3 virus suggests that the enhancement of H77S virus production in the presence of DMAT could be due to nonspecific target effect(s) of this inhibitor and that this nonspecific effect is exclusive with this genotype 1a virus. We do not know which viral factor(s) was affected nonspecifically by DMAT. Certainly, NS2 and NS5A domain III are not the targets of this effect since the two viruses (H77S/J5Ad3 and HJ3-5) which contain the same NS2 and NS5A domain III showed the opposite outcome upon DMAT treatment (Fig. 1B and Fig. 3A). Whatever is responsible for such nonspecific target effect, it seems to be strong enough to negate a small negative effect on H77S virus production by specific CKII inhibition.

### TBCA increases virus production of genotype 1a HCV

**Figure 6:**
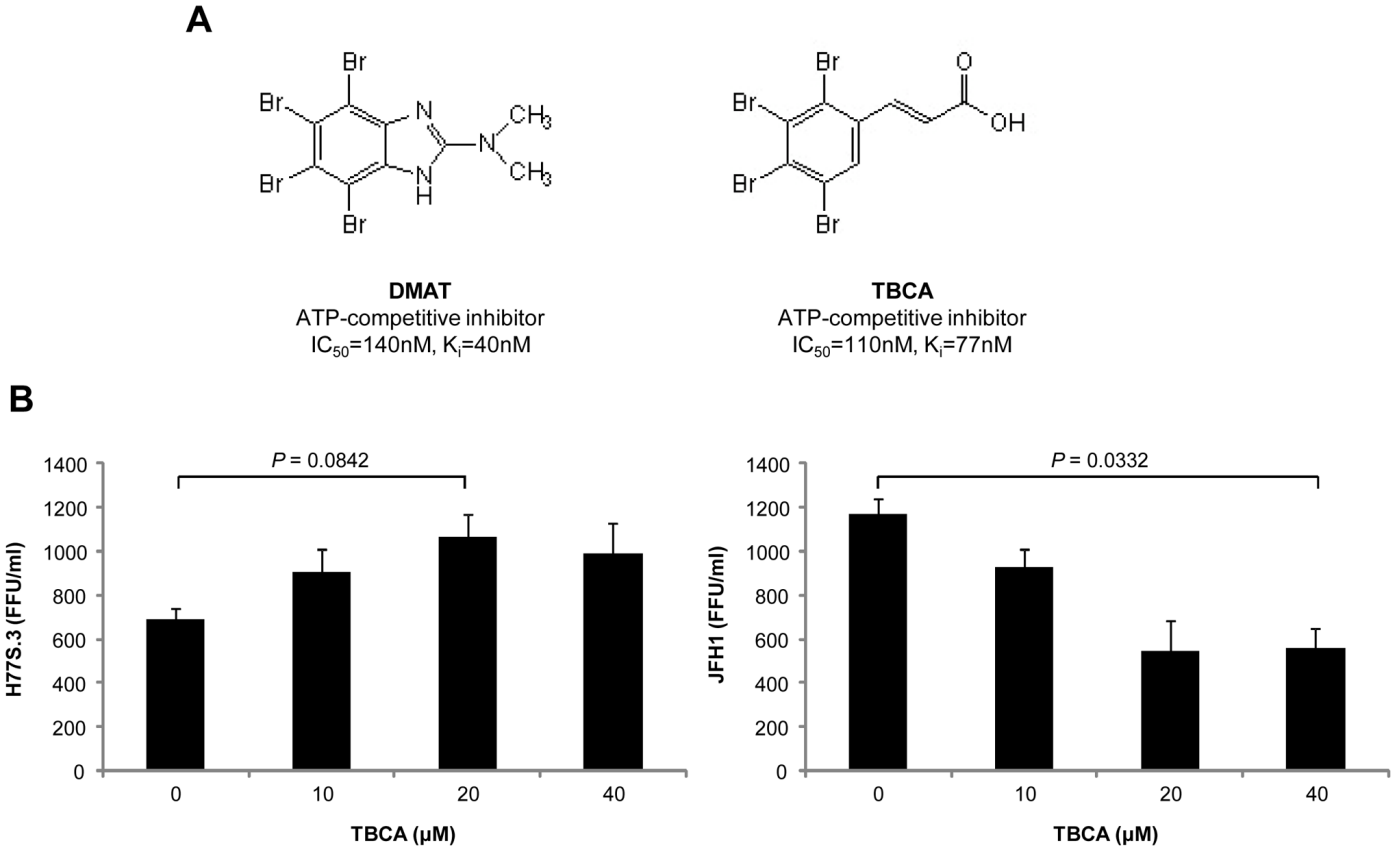
Effect of TBCA, another CKII inhibitor, on the production of H77S.3 and JFH1 virus. (A) Chemical structure of DMAT and TBCA. (B) Following transfection of the HCV RNA, cells were treated with the indicated concentration of TBCA for 48 hours. The media was then replaced with fresh medium (no drug), followed 24 hours later by harvesting of supernatant fluids for virus titration. Means ± S.E. were calculated from duplicate experiments. *P* values were determined from unpaired *t* tests.

Since the nonspecific target effect of DMAT that we observed might be unique with this compound, we tried another CKII inhibitor, (*E*)-3-(2,3,4,5-tetrabromophenyl)acrylic acid (TBCA) (Fig. 6A). Both DMAT and TBCA are compounds derived from TBB (4,5,6,7-tetrabromo-1*H*-benzotriazole), but TBCA has a better selectivity for CKII [27]. Huh7.5 cells were transfected by HCV RNA and 6 hours after transfection, TBCA was added to the culture medium and maintained for 48 hours. Three days after transfection, culture supernatants were collected for virus titration (Fig. 6B). Although even higher concentration of TBCA was required to observe the effect on virus production, very similar results were obtained compared to those of DMAT. H77S.3 virus titer increased but JFH1 virus titer decreased when the concentration of TBCA increased.

Since DMAT inhibits both CKII (IC_50_ = 0.14 µM) and DYRK1A (dual specificity tyrosine-phosphorylation-regulated kinase 1A) (IC_50_ = 0.12 µM) similarly [28], we tested whether DYRK1A is involved in production of HCV as a nonspecific target by siRNA knockdown. However, we found that DYRK1A is not detectably expressed in Huh7 and Huh7.5 cells by immunoblot (data not shown). Lack of expression of DYRK1A in Huh7 cells is also noted in the GeneCards database [29].

### Other 1a/2a intergenotypic chimeras do not support production of infectious particles

Since viral factors other than NS2 and NS5A domain III may be responsible for the nonspecific effect of CKII inhibitors on H77S virus production, we generated other 1a/2a intergenotypic chimeras between H77S.3 and JFH1 virus. We generated H77S.3/J5B (1a/2a intergenotypic chimera containing NS5B of JFH1 in H77S.3 background), JFH1/H5B (1a/2a intergenotypic chimera containing NS5B of H77S.3 in JFH1 background), JFH1/H3 (1a/2a intergenotypic chimera containing NS3 of H77S.3 in JFH1 background), and JFH1/H4AB (1a/2a intergenotypic chimera containing NS4AB of H77S.3 in JFH1 background) since only these chimeras supported HCV RNA replication when they were assessed by GLuc reporter assay among all the possible 1a/2a intergenotypic combinations between H77S.3 and JFH1 proteins [18]. These 4 chimeras were tested by transfecting RNAs in the presence of DMAT, but none could support production of infectious particle although H77S.3/J5B and JFH1/H4AB could produce detectable amount of intracellular core protein (data not shown).

## Discussion

Most of the currently tested antivirals against HCV infection are targeted to viral proteins, specifically NS3 protease (e.g., boceprevir, telaprevir), NS5A (e.g., daclatasvir), and NS5B (e.g., sofosbuvir). However, there are other candidate inhibitors targeting host factors such as cyclophilin (e.g., alisporivir), miR-122 (e.g., miravirsen), and SR-BI (e.g., ITX-5061) [30]. DMAT was shown previously to inhibit specifically infectious genotype 2a HCV production without affecting viral RNA replication [11], and this suggested that CKII inhibitor could be considered as another therapeutic option for HCV antiviral treatment. In fact, CX-4945, a selective CKII inhibitor, has entered human clinical trials although it was for its anti-tumor activity not for antiviral activity [12].

In this study, we tested the same CKII inhibitor (DMAT) to see whether it affects genotype 1a HCV production in the same manner as genotype 2a virus. Surprisingly, it rather increased genotype 1a virus production without affecting viral RNA replication (Figs. 1 and 2). Further analysis of chimeras constructed between H77S.3 and JFH1 viruses did not identify any single viral protein that may be responsible for such genotypic differences. So far, only NS2 and NS5A are known as HCV proteins phosphorylated by CKII [23], [24]. However, the response to DMAT treatment on the chimeras that were tested in this study suggests that there could be other viral protein(s) affected by CKII inhibitors. Perhaps, this genotypic difference comes from combinations of more than 2 viral proteins rather than from any single viral protein. Interestingly, when the HCV proteins expressed in the HCV RNA-transfected Huh7.5 cells were assessed by immunoblot, the abundance of NS3 protein changed in the same manner as those of NS2 and NS5A proteins, which suggests possible combinatorial effect of DMAT on HCV proteins either directly or indirectly. Lack of any single viral protein that is differentially affected by host kinase depending on the HCV genotypes was also observed in another study [18].

Although genotype 1a HCV production was enhanced by nonspecific target effect of CKII inhibitors, genetic inhibition of CKII by siRNA also displayed differences between H77S.3 and JFH1 virus production (Fig. 5). Compared to the effect of CKII knockdown on JFH1 virus production, H77S.3 virus production was affected very slightly, which argues against the idea of pan-genotypic effect of CKII on HCV assembly [11]. Given that the amino acid sequence identity between H77S.3 and JFH1 is only 58% for the entire NS5A and 46% for the NS5A domain III [17], the differences between the two viruses upon CKII inhibition may not be surprising, but the result from this investigation emphasizes the importance of HCV genotype identification in both basic and clinical studies.

The effect of DMAT on H77S.3/4SA (Fig. 4) was specifically surprising because this mutant was defective in virus production before DMAT treatment although its RNA replication was comparable to that of H77S virus [17]. This result suggests that the serine residues that were substituted by alanine are involved in virus assembly rather than in RNA replication and that the block in virus production of 4SA mutant was alleviated by treatment with DMAT. Although the nonspecific target kinase of DMAT was not identified in this study, this 4SA mutant is another good example illustrating a molecular switch model that determines the function of NS5A between viral RNA replication and virus assembly [7], [8]. Since alanine is not a phosphorylatable amino acid, DMAT seems to inhibit phosphorylation of other serine/threonine residue(s) of either viral or host target substrate, which can restore virus assembly of H77S.3/4SA. Whatever the nonspecific target of CKII inhibitors is, this result indicates that phosphorylation plays an important role in regulating HCV viral life cycle.

CKII is a ubiquitously expressed, constitutively active serine/threonine protein kinase, and more than 300 substrates are already known [31]. It has α and α′ catalytic subunits and β regulatory subunits, thus forming a heterotetrameric holoenzyme. Since CKII has been implicated in many diseases and viral infection [32], numerous inhibitors targeting this kinase have been developed [33] and both DMAT and TBCA that were used in this study are TBB-derived, ATP-competitive CKII inhibitors (Fig. 6A). With regard to CKII inhibition, TBCA (IC_50_ = 0.11 µM) is the best among the 3 inhibitors compared to TBB (IC_50_ = 0.50 µM) and DMAT (IC_50_ = 0.14 µM) [27], [28], [34]. TBCA also has the best selectivity for CKII against DYRK1A, which is a potent nonspecific target of CKII inhibitors. For example, IC_50_ of TBCA for DYRK1A is 24.50 µM while those of TBB and DMAT are 0.91 µM and 0.12 µM, respectively [27], [28], [34]. Despite such high selectivity, TBCA treatment of HCV RNA-transfected cells also resulted in differential virus production between H77S.3 and JFH1 (Fig. 6) as was observed in the DMAT treatment (Fig. 1). Lack of expression of DYRK1A in Huh7.5 cells (data not shown) and the result of CKII knockdown experiment (Fig. 5) suggest that kinase(s) other than CKII and DYRK1A is involved in the enhanced genotype 1a HCV production upon chemical inhibition of CKII. Identification of the target that nonspecifically enhanced genotype 1a HCV production in this study awaits further screening of target kinases and may provide a unique mechanistic insight into the pathogenesis of this clinically more important genotype 1a HCV.
